# relax: the analysis of biomolecular kinetics and thermodynamics using NMR relaxation dispersion data

**DOI:** 10.1093/bioinformatics/btu166

**Published:** 2014-04-09

**Authors:** Sébastien Morin, Troels E. Linnet, Mathilde Lescanne, Paul Schanda, Gary S. Thompson, Martin Tollinger, Kaare Teilum, Stéphane Gagné, Dominique Marion, Christian Griesinger, Martin Blackledge, Edward J. d’Auvergne

**Affiliations:** ^1^PROTEO, Université Laval, Québec G1V 0A6, Canada, ^2^International AIDS Society HQ, CH-1202 Geneva, Switzerland, ^3^Department of Biology, University of Copenhagen, DK-2200 Denmark, ^4^Institut de Biologie Structurale, Grenoble F-38027, France, ^5^Astbury Centre for Structural Molecular Biology, University of Leeds, Leeds LS2 9JT, UK, ^6^Institute of Organic Chemistry & CMBI, University of Innsbruck, A-6020, Austria and ^7^NMR-based Structural Biology, Max Planck Institute for Biophysical Chemistry, D-37077 Göttingen, Germany

## Abstract

Nuclear magnetic resonance (NMR) is a powerful tool for observing the motion of biomolecules at the atomic level. One technique, the analysis of relaxation dispersion phenomenon, is highly suited for studying the kinetics and thermodynamics of biological processes. Built on top of the relax computational environment for NMR dynamics is a new dispersion analysis designed to be comprehensive, accurate and easy-to-use. The software supports more models, both numeric and analytic, than current solutions. An automated protocol, available for scripting and driving the graphical user interface (GUI), is designed to simplify the analysis of dispersion data for NMR spectroscopists. Decreases in optimization time are granted by parallelization for running on computer clusters and by skipping an initial grid search by using parameters from one solution as the starting point for another —using analytic model results for the numeric models, taking advantage of model nesting, and using averaged non-clustered results for the clustered analysis.

**Availability and implementation:** The software relax is written in Python with C modules and is released under the GPLv3+ license. Source code and precompiled binaries for all major operating systems are available from http://www.nmr-relax.com.

**Contact:** edward@nmr-relax.com

Biological macromolecules are intricate machines, and their functions are closely related to their motions. These motions can be studied experimentally at the atomic level by nuclear magnetic resonance (NMR) spectroscopy. Many important biological processes occur on the 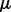
s to ms time scale, and for atoms exchanging between different states, NMR relaxation dispersion can be observed. By studying this exchange process, kinetic and thermodynamic information can be obtained.

For exchanging atoms, their nuclear spin magnetization is described by the Bloch–McConnell equations (McConnell, 1958). Using experimental data, the solution to these equations reveals both populations of the molecular states (thermodynamics) and rates of exchange between them (kinetics). Though the general solution valid for all motions remains intractable, analytic solutions with restricted motions are available and are frequently used. The equations can also be solved numerically.

Two NMR dispersion methods are used for analysing motions: Single, Zero, Double or Multiple Quantum (SQ, ZQ, DQ, MQ) CPMG (Carr and Purcell, 1954; Meiboom and Gill, 1958); or 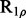 (Deverell *et al.*, 1970). Combined SQ, ZQ, DQ and MQ data will be labelled as Multiple-MQ (MMQ) data. Various models are used to analyse different data and motions. The simplest one is that of no motion (No Rex). For SQ CPMG-type experiments, analytic models include the original Luz and Meiboom (1963) multiple-site fast exchange models (LM63), the Carver and Richards (1972) and population-skewed Ishima and Torchia (1999) 2-site models for most time scales (CR72, IT99) and the Tollinger *et al.* (2001) 2-site very slow exchange model (TSMFK01). The CR72 model has been extended by Korzhnev *et al.* (2004) for MMQ data. For 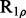-type data, analytic equations include the Meiboom (1961) 2-site fast exchange model for on-resonance data (M61), extended by Davis *et al.* (1994) to off-resonance data (DPL94), and the Trott and Palmer (2002) and Miloushev and Palmer (2005) 2-site models for non-fast and all time scales (TP02, MP05). Different numeric solutions (NS) can be designed for SQ or MMQ data.

Diverse software solutions exist for analysing relaxation dispersion data including CPMGFit (http://www.palmer.hs.columbia.edu/software/cpmgfit.html), cpmg_fit (available on request from Dmitry Korzhnev), CATIA (Hansen *et al.*, 2008), NESSY (Bieri and Gooley, 2011), GUARDD (Kleckner and Foster, 2012), ShereKhan (Mazur *et al.*, 2013) and GLOVE (Sugase *et al.*, 2013). The software relax (d’Auvergne and Gooley, 2008) is a platform for studying molecular dynamics using experimental NMR data, and can be used as a numerical computing environment. Herein, support for relaxation dispersion within relax is presented. Distributed as part of relax, this is the most comprehensive dispersion package supporting the greatest number of dispersion models and NMR data types.

The number of dispersion models supported by relax is extensive (Table 1). This allows for detailed comparisons between modern numeric and traditional analytic approaches. Different user interfaces (UIs) can be used to analyse dispersion data including the prompt, scripting and graphical user interface (GUI). The scripting UI enables the greatest flexibility and allows for most analysis protocols to be replicated. By implementing a novel automated analysis and providing an easy-to-use GUI based on this auto-analysis, the study of dispersion data is much simplified.

**Table 1.** Comparison of model support for different dispersion software

Software	CPMG-type									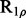 -type					
	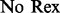	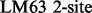	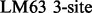	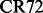	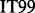	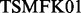	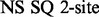	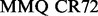	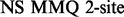	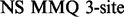	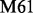	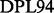	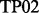	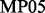	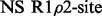	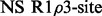
CPMGFit		✓	✓	✓	✓											
cpmg_fit	✓						✓	✓	✓	✓			✓		✓	✓
CATIA							✓									
NESSY	✓	✓	✓	✓												
GUARDD	✓							✓								
ShereKhan		✓		✓			✓									
GLOVE	✓	✓		✓	✓		✓	✓	✓	✓						
relax	✓	✓	✓	✓	✓	✓	✓	✓	✓	✓	✓	✓	✓	✓	✓	✓

The set-up of the auto-analysis includes defining the molecular system, loading the dispersion data directly from peak lists, clustering atoms with the same kinetics, modifying the list of dispersion models and setting up Monte Carlo (MC) simulations for error propagation. Execution involves sequential optimization of the models, fixed model elimination rules to remove failed models and failed MC simulations increasing both parameter reliability and accuracy (d’Auvergne and Gooley, 2006) and a final run whereby Akaike's Information Criterion (AIC) model selection is used to judge statistical significance (Akaike, 1973; d’Auvergne and Gooley, 2003). The optimization is designed for absolute accuracy and robustness, but, as this can take time, it has been parallelized at the spin cluster and the MC simulation level to run on computer clusters using OpenMPI. Three additional methods are used to speed up calculations, all designed to skip the computationally expensive grid search. The first is model nesting—the more complex model starts with the optimized parameters of the simpler. The second is model equivalence—when two models have the same parameters. For example, the CR72 model parameters are used as the starting point for the CPMG numeric models, resulting in a huge computational win. The third is for spin clustering—the analysis starts with the averaged parameter values from a completed non-clustered analysis.

The dispersion analysis in relax is implemented in Python using NumPy and the GUI using wxPython. Optimization using the Nelder–Mead simplex and log-barrier constraint algorithms from the minfx library (https://gna.org/projects/minfx/) removes the need for numerical gradient approximations, which add a second numeric layer to the NS models. Data visualization is via the software Grace.
