# relax: the analysis of biomolecular kinetics and thermodynamics using NMR relaxation dispersion data

**DOI:** 10.1093/bioinformatics/btz397

**Published:** 2019-06-04

**Authors:** Sébastien Morin, Troels E Linnet, Mathilde Lescanne, Paul Schanda, Gary S Thompson, Martin Tollinger, Kaare Teilum, Stéphane Gagné, Dominique Marion, Christian Griesinger, Martin Blackledge, Edward J d’Auvergne

*Bioinformatics*, (2014) doi: 10.1093/bioinformatics/btu166

In the above article funding information was initially omitted. The following funding statement has been added to the article online:

This work was supported by the European Research Council (ERC-StG-2012-311318-ProtDyn2Function)

The author apologises for this error.

